# Determinants of sibling relationships in the context of mental disorders

**DOI:** 10.1371/journal.pone.0322359

**Published:** 2025-04-23

**Authors:** Flavia Lecciso, Chiara Martis, Cristina Maria Del Prete, Paola Martino, Patrizia Primiceri, Annalisa Levante

**Affiliations:** 1 Department of Human and Social Sciences, University of Salento, Lecce, Italy; 2 Lab of Applied Psychology, Department of Human and Social Sciences, University of Salento, Lecce, Italy; 3 Department of Physical Medicine and Rehabilitation, Local Health Service, Lecce, Italy; 4 Office for Inclusion of Individuals with Disability, University of Salento, Lecce, Italy; University of Maribor, SLOVENIA

## Abstract

Many studies have been conducted on sibling relationships to explore the well-being of siblings of persons with mental disorders. In this research project, two mediation models were tested. Model 1 examined whether a sibling’s distress and the quality of the parent-typically developing sibling relationship would mediate the path between sibling-focused parentification and sibling relationships. Model 2 tested the same paths by comparing siblings of persons with mental disorders (n = 262) and siblings of typically developing persons (n = 303). From March to May 2021, 565 siblings of persons with and without mental disorders were asked to fill in an online survey. Aged 19 to 26 years, most of the siblings who participated in the study were older females. Cross-sectional results showed that the lower sibling-focused parentification was, the higher quality sibling relationships were, through the mediating role of lower distress and higher-quality parent-typically developing sibling relationships (Model 1). Siblings of persons with mental disorders reported low-quality sibling relationships (Model 2). Females and young siblings showed high distress. The findings of this study could become instrumental in promoting high-quality sibling relationships and best practices.

## 1. Introduction

Over the past decade, a large number of studies have been conducted to investigate the psychological impact of disabilities on family members. Research on mothers has revealed high levels of stress and depression [1–3], and distress [4–6]. Studies on fathers [7,8] have shown their being overly busy with work and their lower stress levels compared to those of mothers. Research on persons with disabilities has reported both negative outcomes, including anxiety, depression, and negative emotions [9,10], and positive outcomes, such as high levels of self-efficacy, self-esteem, and acceptance of disability as a resource [11–14].

Interestingly, over the past few years, an increasing number of studies have focused on typically developing (TD) siblings. A recent review [15] has revealed mixed findings in this research field, including harmonious [16–19] rather than conflicting relationships [20,21], high levels of anxiety and depression among siblings [22–24], and high levels of sibling-focused parentification, with siblings taking on adult-like responsibilities and caregiving roles [25].

Given the pivotal role that siblinghood plays in individual growth, adjustment, and psychosocial functioning [26–28], understanding the impact of disabilities on sibling relationships seems to be a pressing public health concern.

Studies on sibling relationships have found that the severity of a disability affects the quality of sibling relationships. As it has been pointed out by Rossetti et al. [29], the more pervasive the disorder is (e.g., mental disorders), the lower quality the sibling relationship is. However, research on the connection between mental disorders and sibling relationships has yielded inconsistent findings [30–32]. On the one hand, studies have reported strong emotional sibling relationships [30], with siblings enjoying spending time doing activities together with their brothers or sisters with mental disorders [31]. On the other hand, siblings of persons with mental disorders have reported frustration [30], difficulties in interaction due to the unpredictable behaviour of their brothers or sisters [31], and low levels of closeness [32]. Therefore, sibling relationships can be said to be a construct that should be investigated more thoroughly in this vulnerable target population.

In order to achieve the purpose of this research, empirical studies were analysed that involved siblings of persons with mental disorders and focused on the connection between sibling relationships and both risk and protective determinants. Risk determinants included sibling-focused parentification, distress, and low-quality parent-TD sibling relationships (Section 1.1). The protective determinants considered were perceived social support and perceived benefits of parentification (Section 1.2). Studies on sibling relationships that compared the well-being of siblings of persons with mental disorders and that of siblings of persons without mental disorders were also summarised (Section 1.3).

### 1.1. Risk determinants associated with low-quality sibling relationships

Three risk determinants impacting sibling relationships were considered in this study, that is, sibling-focused parentification, siblings’ distress, and low-quality parent-TD sibling relationships [33].

Parentification is the process through which children and adolescents take on adult-like responsibilities in order to take care of a family member [34–36]. When this sense of responsibility is felt towards a brother or sister, sibling-focused parentification is to be considered a determinant. According to Burton [34], this phenomenon is a form of adultification in which an individual takes on the role of a parent to their brother or sister. Research on siblings of persons with mental disorders [37,38] has revealed that sibling-focused parentification leads to positive relationships being established between TD persons and their brothers or sisters with mental disorders. In other words, the more responsibilities siblings take on that are associated with the challenge and difficulty of taking care of their brothers or sisters with mental disorders (i.e., sibling-focused parentification), the higher quality their sibling relationships are.

The second risk determinant reviewed was distress, which is a combination of anxiety traits, depressive and stress symptoms [39]. A study conducted by Tomeny and colleagues [26] on the relationship between sibling-focused parentification and distress in siblings of persons with mental disorders has revealed a positive direct path between such aspects when only stress, as a distress dimension, is considered an outcome. In other words, the responsibilities associated with sibling-focused parentification are positively related to high levels of stress. However, Tomeny and colleagues [26] did not consider the distress scale. In another study by Tomeny and colleagues [38], the path between sibling-focused parentification and distress was not significant. To date, only one study [38] has been conducted on the link between siblings’ distress and the quality of sibling relationships when siblings of persons with mental disorders are involved. Findings have revealed a negative path between these determinants, as the higher distress is, the lower quality the sibling relationship is.

The quality of the parent-TD sibling relationships was the third risk determinant taken into account in order to investigate the quality of sibling relationships. It is worth noting that this determinant was considered in terms of negative emotions characterising the quality of the parent-TD sibling relationship. As it has been pointed out [40–42], this relationship may be negatively affected by the role of caregiver taken on by the sibling of a person with a disability. More specifically, it has been argued that siblings perceive caregiving as being imposed by parents, which results in harmful and low-quality parent-child relationships. Only a study by Chiu [43] has explored the relationship between parents and TD siblings of persons with mental disorders. The siblings participating in it described their childhood relationship with their parents as being problematic and characterised by ambivalent feelings, due to the little amount of time they spent with their parents [43].

In this study, sibling-focused parentification was posited as a predictor of the quality of sibling relationships through the mediating role of siblings’ distress and the quality of the parent-TD sibling relationship.

### 1.2. Protective determinants predicting sibling relationships

The protective determinants predicting sibling relationships considered in this study were perceived social support and perceived benefits of parentification [33]. In the following paragraphs, a brief description of each determinant will be provided, and empirical studies will be summarised that focus on the connection between each protective determinant and sibling relationships involving siblings of persons with mental disorders.

The first determinant investigated was perceived social support, which is seen as a buffer generating positive emotional experiences [44–46] by protecting individuals from the deleterious effects of stress [45,46], depressive symptoms [47], and adjustment difficulties [48]. Previous research has shown that the sibling relationship between TD siblings and their brothers or sisters with mental disorders benefits from the social support they receive [38,49–51]. Similarly, when social support is provided by parents, high-quality sibling relationships are encouraged [52].

The second determinant examined, perceived benefits of parentification, refers to a TD sibling’s positive thoughts and feelings related to taking on adult-like roles and responsibilities in order to take care of their brothers or sisters [35]. This determinant implies feelings of happiness as a TD sibling takes on the role of caregiver and/or perceives their own family as a team [35]. Evidence [37,53] has shown that high levels of perceived benefits of parentification in siblings of persons with mental disorders are associated with high-quality sibling relationships. Furthermore, the perceived benefits of parentification are connected with a TD sibling’s increased intention to take care of their brother or sister with a disability in adulthood [54].

In light of the aforementioned evidence, both perceived social support and perceived benefits of parentification were included as protective determinants in the model proposed in this study.

### 1.3. Quality of sibling relationships involving siblings of persons with and without mental disorders

Only three studies [55–57] seem to have compared siblings of persons with and without mental disorders , with inconsistent findings . On the one hand, higher closeness [55], lower levels of conflict and rivalry [55], and a higher level of affective quality [56] have been reported by siblings of persons with mental disorders compared to siblings of persons without mental disorders. On the other hand, a study conducted by Doody and colleagues [57] has revealed that siblings of persons with mental disorders experience less warmth in sibling relationships than siblings of persons without mental disorders.

Due to the paucity of information and mixed findings, further research on this topic should be encouraged. For this reason, the serial mediation model used in this study was tested by comparing siblings of persons with mental disorders and siblings of persons without mental disorders.

## 2. Materials and methods

### 2.1. The study

This study aimed to test a previously developed serial mediation model [33] on siblings of persons with mental disorders. The model explored the impact of both siblings’ distress and low-quality parent-TD sibling relationships as risk determinants on the path between sibling-focused parentification as a risk determinant and the quality of sibling relationships as an outcome. Perceived social support and perceived benefits of parentification, as protective determinants, were considered covariates. Results showed a full serial mediation effect. In other words, the higher the level of sibling-focused parentification was, the lower quality the sibling relationship was, through the mediating role of high levels of siblings’ distress and low-quality parent-TD sibling relationships. The higher the level of perceived social support was, the lower the distress experienced by siblings was. Findings also highlighted that perceived benefits of parentification were associated with lower levels of distress and higher-quality parent-TD sibling relationships.

Despite leading to interesting findings, the previous study in which the model was first used focused on siblings of persons with different types of disabilities, including genetic diseases, physical disabilities, multiple disorders, neuropsychiatric disorders, and sensory disabilities, which limited the generalisation of the findings themselves. Furthermore, no control group was included in that study, as the well-being of siblings of TD persons was not investigated. In order to address the limitations of the previous study [33], this study had a twofold aim: (1) to explore the model paths in siblings of persons with mental disorders and (2) compare siblings of persons with mental disorders with siblings of persons without mental disorders.

### 2.2. Study hypotheses and research question

Two hypotheses were formulated and tested considering the health/disability status of the participants’ brothers or sisters, which led to dividing the participants into two groups, i.e., siblings of persons with mental disorders and siblings of persons without mental disorders. Model 1 tested whether: *The level of sibling-focused parentification predicts the quality of sibling relationships through the mediating role of the level of siblings’ distress and the quality of parent-TD sibling relationships* (**HP1**). Model 1 also investigated whether: *The levels of perceived social support and perceived benefits of parentification predict siblings’ distress, the quality of parent-TD sibling relationships, and the quality of sibling relationships* (**HP2**)*.*

A research question (RQ) was developed to explore whether *any differences can be identified in the quality of sibling relationships involving siblings of persons with mental disorders and sibling relationships involving siblings of typically developing persons* (**RQ**).

**Fig 1:**
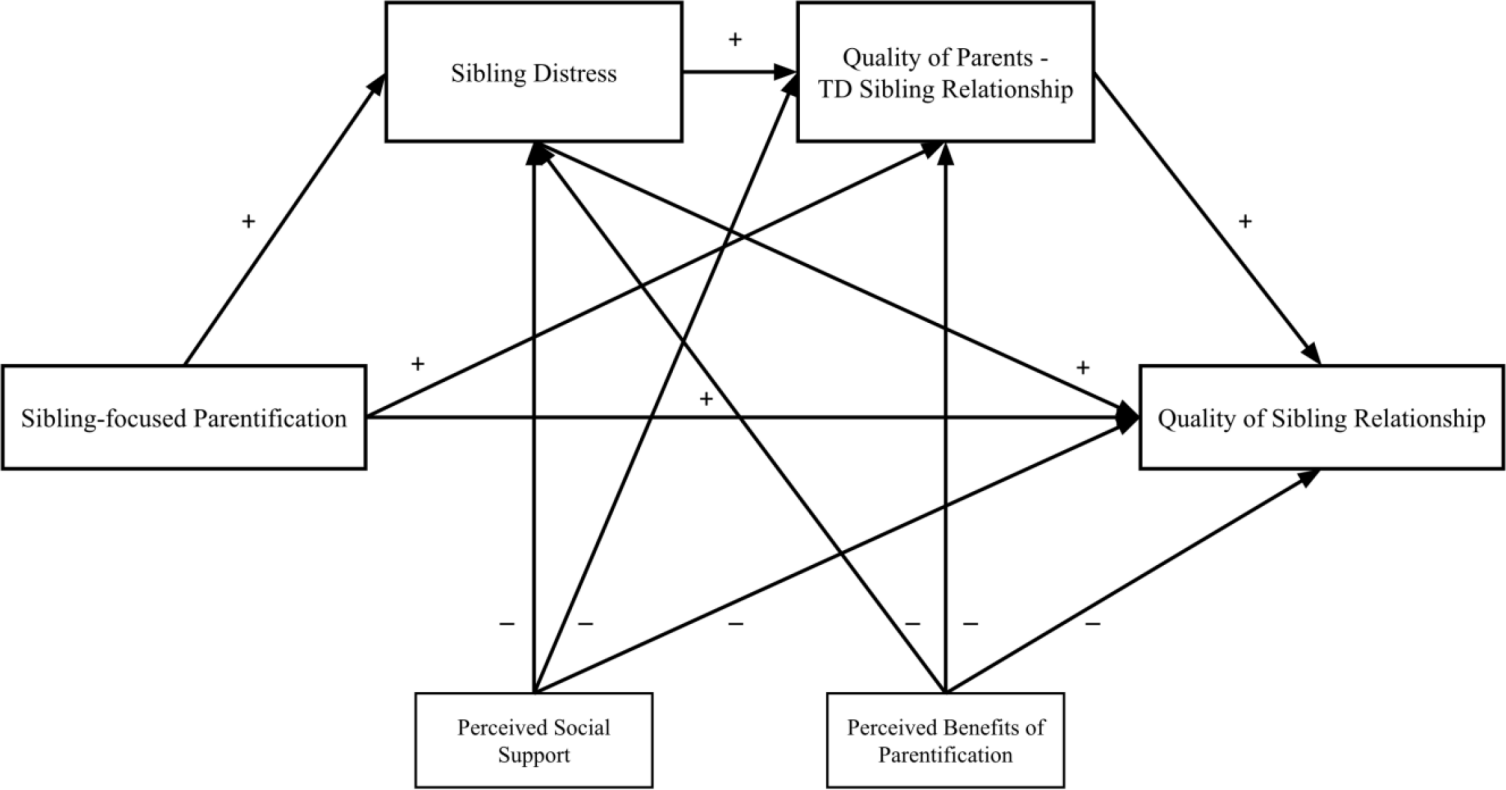
Serial mediation model to be tested.

Fig 1 shows the path directions in the mediation model to be tested.

### 2.3. Methods

#### 2.3.1. Procedure.

This study involved two groups of Italian siblings aged 19–26 years. The first group consisted of siblings of persons with mental disorders, while the second one was made up of siblings of persons without mental disorders. An online survey was imported in Microsoft Forms and distributed from 1 March to 31 May 2021 via both the main social media platforms (e.g., WhatsApp, Instagram, Facebook) and a mailing list of several non-profit organisations associated with a local health service provider – the Local Health Unit in Lecce, Italy. A non-probabilistic method was used to recruit participants. A link to the survey was distributed to members of the organisations involved or via posts on social media (i.e., purposive sampling). Additionally, a message was included in the online survey in order to ask participants to distribute it to their contacts (i.e., snowball sampling). Two inclusion criteria were predefined: (1) being a sibling of a person with or without a mental disorder and (2) being aged 19–26. Exclusion criteria were: (1) having a disability, (2) having a brother or sister with a disability that could not be considered a mental disorder, (3) not falling within the age range examined, and (4) not being fluent in the Italian language.

This research project was approved by the Ethics Committee of the University of Salento (project reference number: 0056300). All the participants were provided with information and signed an online informed consent form prior to their participation in the survey.

### 2.4. Participants

The online survey was filled in by 565 siblings. In Section 2.4.1, a descriptive analysis of the subsample of siblings of persons with mental disorders will be carried out. In Section 2.4.2, the socio-demographic information on the subsample of siblings of persons without mental disorders will be summarised.

#### 2.4.1. Siblings of persons with mental disorders.

The group of siblings of persons with mental disorders consisted of 262 participants [*M*(*SD*) = 24.51(1.12) years]. Most of them were female (n = 247; 94.3%) and older (n = 156; 59.5%) than their brother or sister with a disability. As for education, 3.4% of the siblings in this group had a low level of education, having attended school for up to 8 years, 52.3% of the siblings had an intermediate level of education, having completed up to 13 years of schooling, and 44.3% of the siblings had a high level of education, having studied for 16 years or more. Lastly, 49 (18.7%) participants in this group were in a relationship.

The gender distribution of the brothers or sisters with mental disorders was balanced (*n* = 128; 48.9% female) and their mean age was 21.98 years (SD = 6.83 years). As for their level of education, 12.5% of data were missing, while 35.9% of the brothers or sisters with mental disorders had a low level of education, having attended school for up to 8 years, 49.3% of the brothers or sisters had an intermediate level of education, having completed up to 13 years of schooling, and 2.3% of the brothers or sisters had a high level of education, having studied for 16 years or more.

#### 2.4.2. Siblings of persons without mental disorders.

The group of siblings of persons without mental disorders consisted of 303 participants [*M*(*SD*) = 22.47 (2.29) years]. Most of them were female (*n* = 242; 79.9%) and older (*n* = 157; 51.8%) than their brothers or sisters. As for education, 4.9% of the siblings in this group had a low level of education, having attended school for up to 8 years, 55.4% of the siblings had an intermediate level of education, having completed up to 13 years of schooling, and 39.6% of the siblings had a high level of education, having studied for 16 years or more. Lastly, 28 (9.2%) participants in this group were in a relationship.

The mean age of the brothers or sisters without mental disorders (*n* = 161 females; 53.1%) was 18.45 years (*SD* = 6.32 years). As for their level of education, 0.6% of data were missing, while 29.7% of the brothers or sisters without mental disorders had a low level of education, having attended school for up to 8 years, 41.6% of the brothers or sisters had an intermediate level of education, having completed up to 13 years of schooling, and 28.1% of the brothers or sisters had a high level of education, having studied for 16 years or more.

### 2.5. Measures

#### 2.5.1. Sibling-focused parentification.

Sibling-focused parentification was measured using the Parentification Inventory [35]. The standard self-report parentification questionnaire consists of three scales that assess parent-focused parentification, sibling-focused parentification, and perceived benefits of parentification. However, for the specific purposes of this study, the Parent-Focused Parentification Scale was not included in the online questionnaire administered to participants, as such a scale captures the adult-like roles and responsibilities taken on by an individual in order to take care of their parents. On the other hand, the 7-item Sibling-Focused Parentification and the 3-item Perceived Benefits of Parentification Scales were used.

The Sibling-Focused Parentification Scale captured the adult-like roles and responsibilities taken on by siblings to take care of their brothers or sisters [siblings of persons with mental disorders: *M*(*SD*) = 2.43(.85); α = .84; r > .32; siblings of persons without mental disorders: *M*(*SD*) = 1.94(.73); α = .80; r > .322)]. An example item of this scale was “*I was expected to comfort my siblings when they were sad or having emotional difficulties*”.

The Perceived Benefits of Parentification Scale assessed the positive states of mind associated with parentification [siblings of persons with mental disorders: *M*(*SD*) = 3.33(1.07); *α* = .87; *r* > .670; siblings of persons without mental disorders: *M*(*SD*) = 3.41(1.02); *α* = .88; *r* > .689)]. An example item of this scale was “*I felt appreciated by my family*”. Response options ranged from 1 (“Never True”) to 5 (“Always True”). The two subscales were calculated as the mean of items based on Hooper [35] scoring system, with higher scores indicating higher levels of sibling-focused parentification and more perceived benefits of parentification. For the specific purposes of this study, the English version of the administered items was translated into Italian by two of the authors of this paper.

#### 2.5.2. Siblings’ distress.

Siblings’ distress was measured using the Depression Anxiety Stress Scale (DASS-21; 39). The 21-item self-report questionnaire administered consisted of three scales assessing depression (e.g., “*I felt that I had nothing to look forward to*”), anxiety (e.g., “*I felt I was close to panic*”), and stress (e.g., “*I felt that I was using a lot of nervous energy”*). For the specific purposes of this study, the aggregate score of distress was included in the model [siblings of persons with mental disorders: *M*(*SD*) = 2.06(.61); *α* = .94; *r* > .300; siblings of persons without mental disorders: *M*(*SD*) = 1.97(.59); *α* = .94; *r* > .307)]. Response options ranged from 0 (“Did not apply to me at all”) to 3 (“Applied to me very much, or most of the time”). High scores indicated high levels of siblings’ distress. The Italian version [39] of the measure was used.

#### 2.5.3. Siblings’ perceived social support.

Perceived social support was measured using the Multidimensional Scale of Perceived Social Support [58]. The 12-item self-report questionnaire administered assessed the siblings’ perceived social support from (1) family (e.g., “*I can talk about my problems with my family*”), (2) friends (e.g., “*I have friends with whom I can share my joys and sorrows*”), and (3) significant other (e.g., “*There is a special person with whom I can share joys and sorrows*”). Response options ranged from 1 (“Very Strongly Disagree”) to 7 (“Very Strongly Agree”). The total score was calculated as the average of all the items, with high scores indicating high levels of perceived social support [siblings of persons with mental disorders: *M*(*SD*) = 5.34(1.18); *α* = .90; *r* > .307; siblings of persons without mental disorders: *M*(*SD*) = 5.59(1.09); *α* = .91; *r* > .300)]. For the specific purposes of this study, the Italian version (Di Fabio & Busoni, 2008) of the measure was used.

#### 2.5.4. Quality of the sibling relationship and quality of the parent-TD sibling relationship.

Two measures were developed and previously used [33] by the authors of this paper to assess the quality of sibling relationships and parent-TD sibling relationships. Both measures aimed at assessing the negative aspects characterising the relationship between participants and their brothers or sisters with and without disabilities and the relationship between participants and their parents. More specifically, participants were asked to report how often they had to deal with incomprehension, social withdrawal, aggressive behaviour, too much protection, shame, guilt, too much responsibility, and indifference in their relationships with siblings and parents. Response options ranged from 1 (“Never”) to 4 (“Always”). Two scores were calculated as the average of all the items, with high scores indicating low-quality relationships between participants and their brothers or sisters with disabilities [siblings of persons with mental disorders: M(*SD*) = 2.08(.50); *α* = .70; *r* > .322; siblings of persons without mental disorders: *M*(*SD*) = 1.75(.45); *α* = .70; *r* > .300)] and between participants and their parents [siblings of persons with mental disorders: *M*(*SD*) = 2.08(.55); *α* = .75; *r* > .309; siblings of persons without mental disorders: *M*(*SD*) = 1,98(.52); *α* = .78; *r* > .301)].

#### 2.5.5. Covariates.

Several covariates were included in the serial mediation models developed. In particular, perceived social support, perceived benefits of parentification, type of sibship (younger *vs* older sibling), and gender were included in both mediation models. In Model 2, the health/disability status of the participants’ brothers or sisters (siblings of persons with mental disorders *vs* siblings of persons without mental disorders) was also considered.

### 2.6. Data analysis plan

Statistical analyses were performed using SPSS version 25 [59]. No missing data imputation techniques were used, due to data items being made mandatory. Preliminary parametric and non-parametric analyses were carried out. Pearson’s *r* correlations were computed. The two serial mediation models were tested by using Model 6 and 5000 bootstrap samples for coefficients. In both models, sibling-focused parentification was the predictor (X), quality of the sibling relationship was the outcome (Y), siblings’ distress (M1) and quality of the parent-TD sibling relationship (M2) were the mediators.

## 3. Results

### 3.1. Preliminary analyses

An analysis of the data on siblings of persons with mental disorders revealed no significant differences in terms of the siblings’ gender and type of sibship.

Conversely, non-parametric analyses of the data on siblings of persons without mental disorders showed that female participants reported higher levels of distress [*M*(*SD*) = 2.01(.60)] than their male counterparts [*M*(*SD*) = 1.82(.53); *U* = 6057.5; *p* = .03)]. As for the type of sibship, findings revealed that younger siblings [*M*(*SD*) = 2.08(.63)] of persons without mental disorders showed higher levels of distress [*t*(301) = −3.250; *p* = .001] than older ones. Similarly [*t*(301) = −2.484; *p* = .014], younger [*M*(*SD*) = 2.06(.55)] siblings of persons without mental disorders reported a poorer relationship with their parents compared to older siblings [*M*(*SD*) = 1.91 (.49)].

### 3.2. Correlation analyses

Tables 1 and 2 show the correlations between participants. Data on siblings of persons with mental disorders (Table 1) revealed that sibling-focused parentification was positively associated with siblings’ distress, low-quality parent-TD sibling relationships, and low-quality sibling relationships. Sibling-focused parentification was also negatively correlated with perceived social support. No significant association between sibling-focused parentification and perceived benefits of parentification was found.

**Table 1:** Correlations between study variables of the siblings of persons with a mental disorder.

	(1)	(2)	(3)	(4)	(5)
Sibling-focused parentification	.323***	.236***	.171**	-.105 (.089)	-.134*
Siblings’ distress (1)		.505***	.396***	-.440***	-.444***
Quality of parents-TD sibling relationship (2)			.602***	-.638***	-.515***
Quality of SR (3)				-.367***	-.240***
Perceived BP (4)					.689***
Perceived SS (5)					–

TD = typically developing; SR = sibling relationship; BP = perceived benefits of parentification; SS = perceived social support.

*** *p* < 0.001; ** *p* < 0.010; * *p* < 0.050.

Data on siblings of persons without mental disorders (Table 2) showed that sibling-focused parentification was positively correlated with siblings’ distress, low-quality parent-TD sibling relationships, and low-quality sibling relationships. No significant correlations were found between sibling-focused parentification, perceived social support, and perceived benefits of parentification.

**Table 2:** Correlations between study variables of the siblings of persons without a mental disorder.

	(1)	(2)	(3)	(4)	(5)
Sibling-focused parentification	.249***	.242***	.185***	-.065 (.259)	-.032 (.578)
Siblings’ distress (1)		.463***	416***	-.462***	-.394***
Quality of parents-TD Sibling relationship (2)			.602***	-.586***	-.439***
Quality of SR (3)				-.397***	-.367***
Perceived BP (4)					.607***
Perceived SS (5)					–

TD = typically developing; SR = sibling relationship; BP = perceived benefits of parentification; SS = perceived social support.

### 3.3. Main serial mediation model

#### 3.3.1. Model 1: Hypothesis 1 and hypothesis 2.

**Fig 2:**
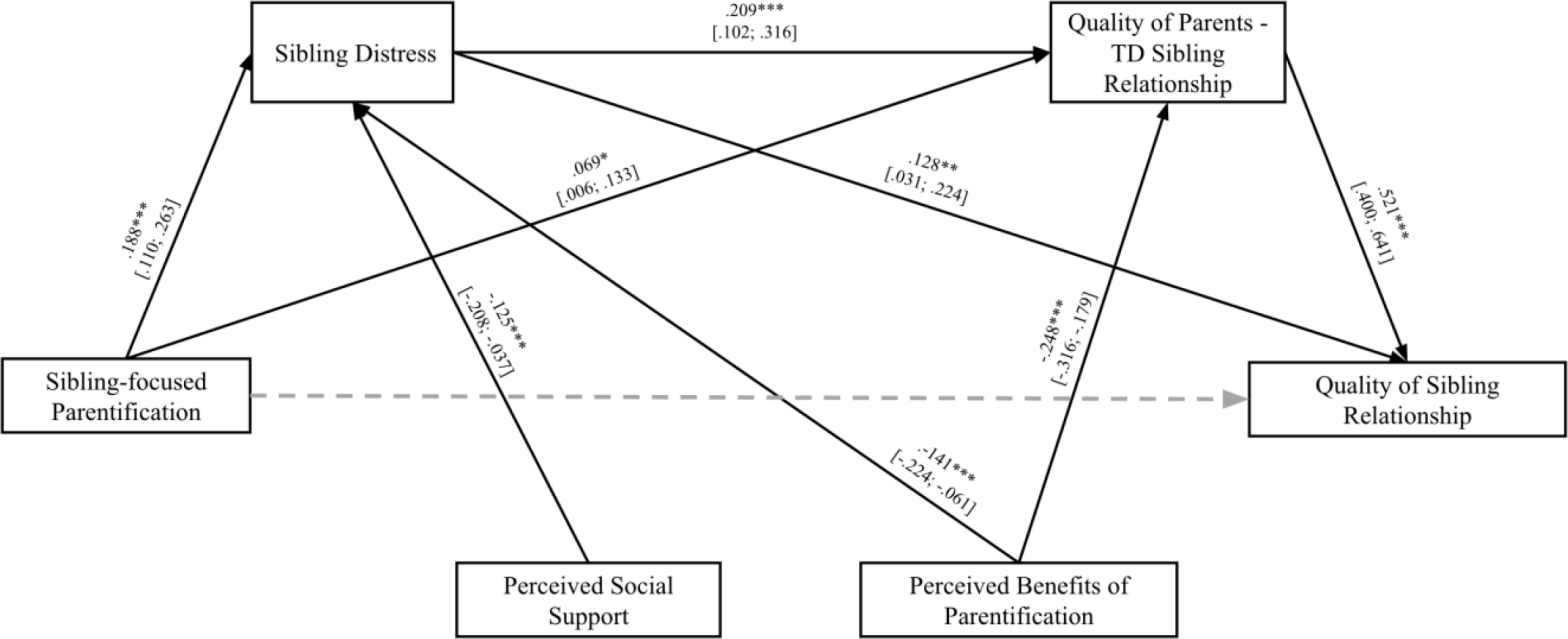
Model 1. * p < 0.05; ** p < 0.01; ***p < 0.001. Non-significant paths were displayed by dotted lines.

Model 1 was tested on a sample of siblings of persons with mental disorders. Findings are reported in Table 3 and Fig 2.

**Table 3:** Betas coefficients, standard errors, p-values, and bootstrap confidence intervals of the serial mediation of Model 1.

Path		Beta	SE	*p-value*	95% Bootstrap CI	
					BootLLCI	BootULCI
Sibling-focused Parentification →	Distress	.188	.038	<.001	.110	.263
	Quality of Parents-Sibling Relationship	.069	.033	.028	.006	.133
	Quality of SR	.005	.031	.865	-.055	.065
Distress →	Quality of Parents-Sibling Relationship	.209	.209	<.001	.102	.316
	Lower Quality of SR	.128	.127	.011	.031	.224
Quality of Parents-Sibling Relationship →	Quality of SR	.521	.522	<.001	.400	.641
**Indirect paths**						
Sibling-focused Parentification → Distress Quality of SR		.041	.018	–	.009	.078
Sibling-focused Parentification → Quality of Parents-Sibling Relationship → Quality of SR		.061	.031	–	.005	.126
Sibling-focused Parentification → Distress → Quality of Parents-Sibling Relationship → Quality of SR		.035	.012	–	.015	.060

SR = sibling relationship.

The full effect was significant [*F*_(6,255)_ = 8.367; *R* = .406; *p* < .001]. The direct path between sibling-focused parentification and quality of sibling relationship was not significant (*β* = .005; *p* = .865), while the paths between sibling-focused parentification and both siblings’ distress (*β* = .188; *p* < .001) and parent-TD sibling relationship (*β* = .069; *p* = .028) were significant. Findings showed that parentification and the responsibilities that siblings take on to take care of their brothers or sisters with mental disorders predict low-quality sibling relationships through the mediating role of higher distress and lower-quality parent-TD sibling relationships. Results supported Hypothesis 1. The paths between siblings’ distress and both quality of sibling relationship (*β* = .128; *p* = .011) and quality of parent-TD sibling relationship (*β* = .209; *p* > .001) were also significant. This suggested that high distress might lead siblings of persons with mental disorders to experience more negative aspects in their relationships with both their brothers or sisters with disabilities and their parents. The path between quality of parent-TD sibling relationship and quality of sibling relationship was significant (*β* = .521; *p* < .001). In other words, low-quality relationships between siblings and parents lead to lower-quality sibling relationships.

Three mediating effects were found. The first one was related to the mediating role of siblings’ distress: sibling-focused parentification was significantly associated with siblings’ distress, which, in turn, was significantly correlated with the quality of the relationship with a brother or sister with a mental disorder. The second indirect effect was related to the mediating role of the parent-TD sibling relationship: sibling-focused parentification was significantly associated with low-quality parent-TD sibling relationships, which, in turn, was significantly correlated with the quality of the sibling relationship. Finally, the third indirect effect was related to the role of siblings’ distress and the quality of the parent-TD sibling relationship in the correlation between sibling-focused parentification and the quality of the sibling relationship.

Results partially supported Hypothesis 2. More specifically, findings showed that the siblings’ perceived social support only impacted siblings’ distress. In other words, the lower the level of social support perceived by siblings of persons with mental disorders was, the higher their levels of distress were. The paths between perceived social support, quality of parent-TD sibling relationship, and quality of sibling relationship were not significant. Results highlighted that the perceived benefits of parentification only impact siblings’ distress and parent-TD sibling relationships. This showed that experiencing positive states of mind due to growing up with a brother or sister with a mental disorder leads siblings to perceive low levels of distress and establish high-quality relationships with their parents.

No significant effects of the other covariates – type of sibship, siblings’ gender, and gender of the brother or sister with a mental disorder – were found.

#### 3.3.2. Research question: Model 2.

**Fig 3:**
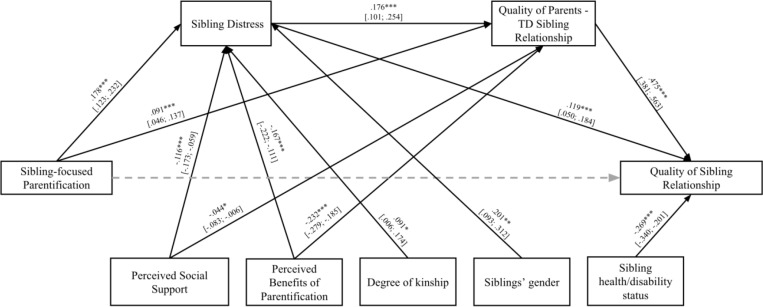
Model 2. * p < 0.05; ** p < 0.01; *** p < 0.001. Non-significant paths were displayed by dotted lines.

Model 2 was tested on siblings of persons with mental disorders and siblings of persons without mental disorders. Findings are reported in Table 4 and Fig 3.

**Table 4:** Betas coefficients, standard errors, p-values, and bootstrap confidence intervals of the serial mediation of Model 2.

Path		Beta	SE	*p*-value	95% Bootstrap CI	
					BootLLCI	BootULCI
Sibling-focused Parentification →	Distress	.178	.028	<.001	.123	.232
	Quality of Parents-Sibling Relationship	.091	.023	<.001	.046	.137
	Lower Quality of SR	.005	.022	.807	-.038	.047
Distress →	Quality of Parents-Sibling Relationship	.176	.039	<.001	.101	.254
	Quality of SR	.119	.034	<.001	.050	.184
Quality of Parents-Sibling Relationship →	Quality of SR	.475	.046	<.001	.381	.563
**Indirect paths**						
Sibling-focused Parentification → Distress → Quality of SR		.035	.011	–	.014	.059
Sibling-focused Parentification → Quality of Parents-Sibling Relationship → Quality of SR		.071	.020	–	.033	.113
Sibling-focused Parentification → Distress → Quality of Parents-Sibling Relationship → Quality of SR		.024	.007	–	.013	.039

SR = sibling relationship.

The full effect was significant [*F*_(7,557)_ = 36.013; *R* = .558; *p* < .001]. Overall, as it was for Model 1, the direct path between sibling-focused parentification and quality of sibling relationship was not significant (*β* = .005; *p* = .807), while the paths between sibling-focused parentification and both siblings’ distress (*β* = .178; *p* < .001) and parent-TD sibling relationship (*β* = .091; *p* < .001) were significant. The paths between siblings’ distress and both quality of sibling relationship (*β* = .119; *p* < .001) and quality of parent-TD sibling relationship (*β* = .176; *p* > .001) were also significant. Furthermore, the path between quality of parent-TD sibling relationship and quality of sibling relationship was significant (*β* = .475; *p* < .001).

Three mediating effects were found also in this case. Firstly, sibling-focused parentification was significantly associated with siblings’ distress, which, in turn, was significantly correlated with the quality of sibling relationships. Secondly, sibling-focused parentification was significantly associated with low-quality parent-sibling relationships, which, in turn, was significantly correlated with the quality of the sibling relationship. Finally, the third indirect effect was related to the role of siblings’ distress and the quality of the parent-TD sibling relationship on the path between sibling-focused parentification and quality of the sibling relationship.

Significant correlations between covariates and both mediators and outcomes were found. Results showed a negative association between perceived benefits of parentification and perceived social support and the two mediators. Furthermore, results highlighted that younger and female siblings reported higher levels of distress than their older and male counterparts. Finally, siblings of persons with mental disorders reported lower-quality sibling relationships than siblings of persons without mental disorders.

## 4. Discussion

This study tested a previously used [33] serial mediation model on siblings of persons with and without mental disorders. Such a mediation model was retested on siblings of persons with mental disorders (Model 1), and paths were explored by comparing siblings of persons with mental disorders and siblings of persons without mental disorders (Model 2).

Model 1 was tested on siblings of persons with mental disorders, with results confirming the findings of the previous study on siblings of persons with different disabilities [33] in which the model was first used. Therefore, findings supported Hypothesis 1 and confirmed the mediating role of distress and quality of the parent-TD sibling relationship. More specifically, the more adult-like duties and responsibilities were taken on by siblings (i.e., sibling-focused parentification), the lower the quality of sibling relationships was, through the mediating role of higher levels of distress and lower-quality parent-TD sibling relationships (HP1).

Despite being preliminary, results seemed to be encouraging and paved the way for two key considerations. Firstly, the model showed how sibling-focused parentification is a significant predictor for low-quality sibling relationships, high levels of distress, and low-quality parent-TD sibling relationships. Consequently, siblings’ experiences of caregiving should be investigated more thoroughly. A more comprehensive analysis of siblings’ perception, experiences, and feelings related to parentification would lead to identifying the difficulties faced and perceived by siblings that may affect their well-being and emotional relationships with family members. A second key consideration should be made on the paths between the two mediators: distress and both low-quality parent-TD sibling relationships and quality of the sibling relationship. The simultaneous and negative impact of these two mediators shows that they may be instrumental in implementing individual and family intervention programmes. Nevertheless, in order to better understand what aspects of each determinant affect the quality of sibling relationships, more studies on siblings of persons with mental disorders are needed.

When the role of perceived social support and perceived benefits of parentification were explored in the model on siblings of persons with mental disorders, HP2 was partially supported. Results were consistent with findings from other studies on siblings of persons with mental disorders (e.g., autism; [60–62]) and other disabilities (e.g., cancer or chronic illness; [63,64]), as they showed that perceived social support served as a buffer only in reducing deleterious outcomes in terms of distress. This means that sharing their own experience of caregiving and talking about their feelings and/or problems with friends, family members, and/or significant others may help siblings to reduce their stress, anxiety, and depressive symptoms. Findings revealed that the role played by perceived benefits of parentification, as a determinant, served as a buffer for siblings’ distress and low-quality parent-TD sibling relationships. Results were consistent with findings from other studies on siblings of persons with mental disorders (i.e., autism; [38]), as they highlighted that when siblings feel appreciated for their caregiving role and perceive that family members are close to each other, they are less distressed, which benefits the quality of parent-TD sibling relationships. Therefore, results showed that these two protective determinants should not be underestimated and/or ignored when intervention programmes aimed at siblings are developed. Similarly, Lazarus and Folkman’s [65] Model of Stress and Coping should be considered a useful tool in this field. For instance, training programmes may be designed so as to help siblings to become more aware of their resources (e.g., proactive coping and/or acceptance of their brothers’ or sisters’ disabilities) and foster their positive state of mind when growing up with, and taking care of, brothers or sisters with disabilities.

Model 1 revealed non-significant paths between siblings’ gender and type of sibship on the mediators and the outcome. As for gender, non-significant results may be related to unbalanced gender distribution. When the type of sibship was considered, young and older siblings reported no differences in distress, quality of parent-TD sibling relationships, and sibling relationships. Such a lack of difference may be explained by the fact that siblings of any age were equally involved in taking care of their brothers or sisters with mental disorders. However, as these individual factors may be critical in identifying the most vulnerable members (female *vs* male siblings; younger *vs* older siblings) of the sibling population, further research in these fields seems to be essential.

The second main purpose of this study was to compare siblings of persons with mental disorders and those of persons without mental disorders (Model 2). Model 2 supported Model 1 and previous results [33]. Therefore, also when siblings of persons without mental disorders were considered, the responsibilities and duties they took on in order to take care of their brothers or sisters (i.e., sibling-focused parentification) predicted low-quality sibling relationships through the mediating role of higher distress and lower-quality parent-TD sibling relationships. The research question formulated aimed to explore whether any difference could be found in the quality of sibling relationships involving the two samples of siblings recruited. Findings mainly showed that siblings of persons with mental disorders reported lower-quality sibling relationships than siblings of persons without mental disorders. Although results were consistent with research by Doody [57], they were inconsistent with findings from other studies [55,56] which revealed that siblings of persons with mental disorders reported higher-quality sibling relationships than siblings of persons without mental disorders. Several factors may explain such mixed results, including the overwhelming responsibilities taken on by siblings who take care of their brothers or sisters with disabilities and/or the pervasive nature of the mental disorder considered. Therefore, heterogeneous findings suggest that further studies are needed so as to explore the sources that are mainly involved. Model 2 also confirmed that perceived social support and perceived benefits of parentification play a protective role in decreasing siblings’ distress and enhancing parent-TD sibling relationships. In conclusion, in this aggregate sample consisting of siblings of persons with and without mental disorders, siblings’ gender and type of sibship were sources of distress, with female and younger siblings being the most distressed. This prompted further reflection. As for siblings’ gender, the unbalanced gender distribution of the participants should lead to interpreting results cautiously. On the one hand, taking care of a brother or sister (with or without a disability) may be described as a mainly female phenomenon only due to the hyper-involvement of female participants in this study, which is focused on psychological determinants particularly affecting women. On the other hand, findings may be interpreted by making reference to the Social Role Theory [66], which argues that women are more naturally inclined to undertake caring tasks both in the presence and absence of family members with disabilities. This behavioural attitude may reflect sociocultural gender stereotypes that women internalise and self-impose [67]. When the Mediterranean culture, and the Italian way of life in particular, is considered, it can be said that women perceive caring as a moral duty [68,69]. Such cautions interpretation of findings highlights the need for future gender-specific studies. Additionally, further research should be conducted to investigate the type of sibship, in order to better understand the difference in the psychological experience of younger and older siblings, regardless of their brothers’ or sisters’ disabilities.

## 5. Implications and limitations

In conclusion, this study aimed to expand the literature on the impact of sibling-focused parentification on the quality of sibling relationships, by considering the serial role of siblings’ distress and the quality of parent-TD sibling relationships. The findings of this study could be used to conduct future research and develop intervention programmes that may foster high-quality sibling relationships. According to Bowen [70], a family is a unit whose dynamics and well-being are influenced by all the members of the family system. Therefore, any intervention programmes should involve both siblings and their parents, which would help to investigate the sources of the above-mentioned determinants and promote protective factors.

As the impact of disability on the functioning of family members may also be related to the type and severity of disability itself [71,72], future research should further examine the serial mediation model on siblings of persons with mental disorders, so as to validate it and expand knowledge of the role of sibling-focused parentification, individual factors (i.e., gender and sibship), environmental resources (i.e., perceived social support), and personal resources (i.e., perceived benefits of parentification) in the quality of sibling relationships. Further studies could test the model on other sibling populations, so as to pave the way for new research opportunities and lead to a better understanding of the sources of quality sibling relationships. Moreover, the mediation model could be examined at different developmental stages, in order to better explore the role played by the determinants involved.

The clinical implications of this study may be beneficial to the development of intervention programmes aimed at reducing risk factors and promoting protective ones. More specifically, results revealed that an increase in perceived social support and awareness of the perceived benefits of parentification may reduce siblings’ distress and foster quality parent-TD sibling relationships. Intervention programmes could focus on facilitating communication on the disability of one’s brother or sister, and/or the difficulties associated with a sibling’s caring role, and/or concerns about the future.

Although findings were encouraging, three limitations should be highlighted. Firstly, the gender distribution in both subsamples of siblings was unbalanced. Secondly, the models were developed from cross-sectional data. Longitudinal studies should be carried out in order to provide further data that may validate the direction and magnitude of the paths. Finally, the narrow age range of the sample limits the generalisation of the findings, although the participants involved fell within the same age range of those recruited in a previous study. The age range of the sample also meant that participants were characterised by five specific features [73]: identity exploration (i.e., the individual decides who they are), instability (e.g., in terms of living in a certain place), self-focus (i.e., the individual is focused on their needs and wants), feeling in-between (i.e., the individual takes on their duties, despite not perceiving themselves as an adult), and possibility (i.e., the individual thinks they will have more opportunities than their parents). Therefore, in light of the challenging duties related to taking care of a brother or sister with a mental disorder [74–76], further studies should explore a sibling’s experience at different developmental stages, in order to expand knowledge of the critical determinants affecting sibling relationships.

In conclusion, this study aimed to provide recommendations to guide future research and develop intervention programmes. Best practices were highlighted that may expand knowledge of determinants affecting the quality of sibling relationships, in both the presence and absence of mental disorders, with the improvement of psychological well-being being promoted within the whole family.

## Supporting information

S1 File Comparative samples. (XLSX)

S2 File Note regarding the dataset. (DOCX)
