# Supplementary material for: Determinants of sibling relationships in the context of mental disorders
Source: PLoS One. 2025 Apr 23;20(4):e0322359. doi: 10.1371/journal.pone.0322359 (PMC12017835; doi:10.1371/journal.pone.0322359)
Supplement: S2 File — (DOCX) [file pone.0322359.s002.docx]

**Note regarding the dataset entitled “Comparative samples”.**

**Socio-demographic features:**

health/disability status: 0 = siblings of persons with mental disorders; 1 = siblings of persons without mental disorders.

gender_brother/sister with or without disability: 0 = males; 1 = females.

gender sibling: 0 = males; 1 = females.

age range sibling: 2 = 19-26 years.

sibship: 1 = older; 2 = younger.

**Measures:**

*Parentification inventory*:

PI_1-PI_7: items assessing the sibling-focused parentification.

P_8-PI_10: items assessing the perceived benefit of parentification.

Scoring: from 1 = Never True … 5 = Always True.

*Depression, Anxiety, Stress Scale*:

DASS_1-DASS_21: items evaluating the distress.

Scoring: from 0 = Did not apply to me at all … 3 = Applied to me very much, or most of the time.

*Multidimensional Scale of Perceived Social Support*:

MSPSS_1, MSPSS_2, …MSPSS_12: these cells reported the raw data regarding the Multidimensional Scale of Perceived Social Support questionnaire.

Scoring: from 1= very strongly disagree… 7 = very strongly agree

*Quality of the Sibling Relationship and Quality of the Parent-TD Sibling Relationship*

emo_neg_par_1: incomprehension

emo_neg_par_2: social withdrawal

emo_neg_par_3: aggressive behaviour

emo_neg_par_4: too much protection

emo_neg_par_5: shame

emo_neg_par_6: guilt

emo_neg_par_7: too much responsibility

emo_neg_par_8: indifference

emo_neg_sibling_1: incomprehension

emo_neg_sibling_2: social withdrawal

emo_neg_sibling_3: aggressive behaviour

emo_neg_sibling_4: too much protection

emo_neg_sibling_5: shame

emo_neg_sibling_6: guilt

emo_neg_sibling_7: too much responsibility

emo_neg_siblign_8: indifference

Scoring: from 1 = Never … 4 = Always.
